# Primary Bone Lymphoma of the Scapula

**DOI:** 10.3390/hematolrep16010011

**Published:** 2024-02-28

**Authors:** Josip Lovaković, Inga Mandac Smoljanović, Andro Matković, Tomislav Smoljanović

**Affiliations:** 1 Department of Surgery, University Hospital Center Zagreb, 10 000 Zagreb, Croatia; 2 Department of Internal Medicine, University Hospital Merkur, School of Medicine, University of Zagreb, 10 000 Zagreb, Croatia; inga.mandac@kb-merkur.hr; 3 Clinical Department for Diagnostic and Interventional Radiology, University Hospital Merkur, 10 000 Zagreb, Croatia; andro.matkovic@student.mef.hr; 4 Department of Orthopaedic Surgery, University Hospital Center Zagreb, School of Medicine, University of Zagreb, 10 000 Zagreb, Croatia; tsmoljan@kbc-zagreb.hr

**Keywords:** primary bone lymphoma, scapula, diffuse large B-cell lymphoma, immunochemotherapy, radiotherapy

## Abstract

Primary bone lymphoma of the scapula is a rare tumor that usually causes local pain. The presented patient suffered for two years from paresthesia, tingling, numbness, and edema of the little and ring fingers. The 45-year-old man underwent several radiological and neurological assessments of the palm, elbow, and neck before radiographs revealed a tumor of the left shoulder. Once diffuse large B-cell lymphoma was confirmed, immunochemotherapy with rituximab, cyclophosphamide, doxorubicin, vincristine, and methylprednisolone (R-CHOP) started. The treatment was accompanied by antiviral treatment with lamivudine due to positive hepatitis B virus serology, specifically anti-HBs (hepatitis B surface) antibody, total anti-HBc (hepatitis B core) antibody, and anti-HBe (hepatitis B e antigen) antibody, together with bisphosphonate treatment for the prevention of bone resorption. Once immunochemotherapy was finished, the treatment was supplemented by radiotherapy of the shoulder. After more than three years of remission, the patient had an ischemic stroke manifesting with right-sided hemiparesis. Following physical therapy, the patient is currently in the process of evaluation for thrombophilia, as well as further cardiac assessment due to the positive transcranial Doppler bubble test, setting high suspicion for the presence of patent foramen ovale.

## 1. Introduction

Primary non-Hodgkin lymphoma of the bone is a rare subtype, accounting for less than 1% of non-Hodgkin lymphomas (NHLs) and approximately 5% of all primary extranodal NHLs [1]. We present an uncommon case significant for the localization and clinical presentation of the tumor, the patient’s comorbidities, and the specificity of the therapeutic approach.

## 2. Case Report

**Figure 1:**
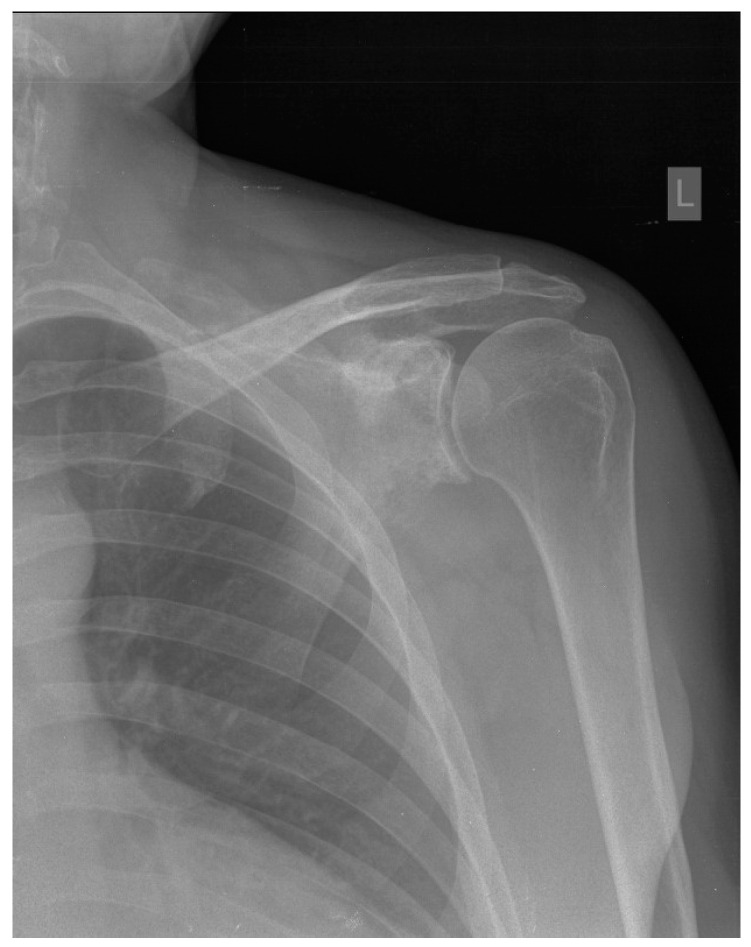
X-rays, anteroposterior (AP) view of the left shoulder. Multiple areas of decreased bone mineralization and multifocal osteolytic lesions are seen in the left scapula, with an area of breached cortical bone in the inferior part of the scapular neck.

**Figure 2:**
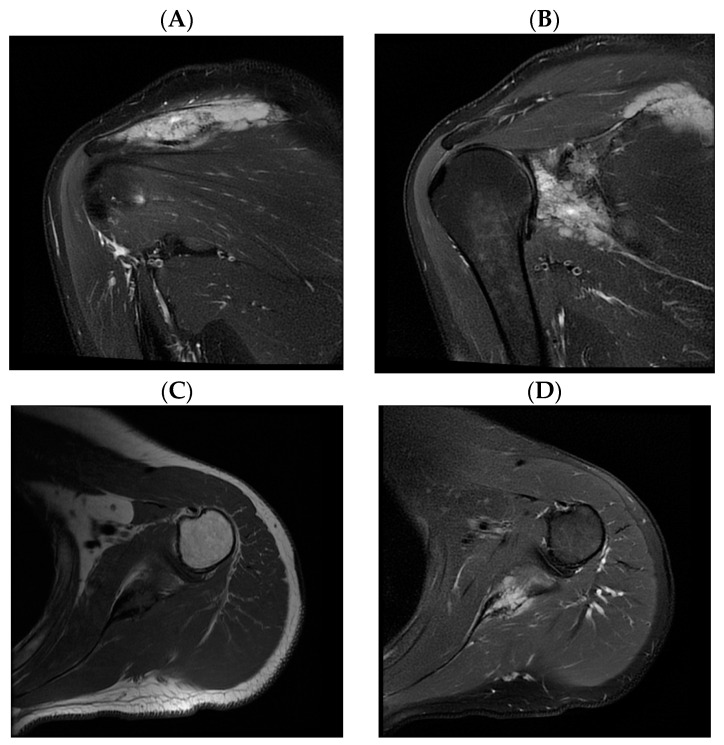
Magnetic resonance imaging (MRI) of the left shoulder. (**A**,**B**) Coronal short tau inversion recovery sequence images show extensive osteolytic, partly multilocular scapular lesions involving the lateral corpus, coracoid process, acromion, glenoid, and spina with periosteal reaction and destruction of cortical bone with extraosseous spread to surrounding soft tissue. No signs of infiltration of the surrounding muscles. (**C**) Axial T1-weighted and (**D**) axial proton density-weighted fat-saturated images show no signs of infiltration of the brachial plexus. The shortest distance between the distal part of the brachial plexus and the tumor is 33 mm. The rotator cuff tendons and the long head of biceps tendon are unremarkable. No signs of glenohumeral joint effusion or significant labral abnormalities.

A 45-year-old patient reported paresthesia, tingling, numbness, and edema of the little and ring fingers of the left hand in mid-2016. Because of inconspicuous clinical presentation, the patient was sent to physical therapy. After that failed, he was referred to a neurologist in August 2016. Neurological examination found a weakened left-hand grip together with a weakened small finger abduction and opposition. Also, a positive Tinel sign of the left hand was observed, and the clinical diagnosis of cubital tunnel syndrome was established. Cervical spine and left elbow X-rays were performed, together with electromyoneurography (EMNG) of the arms. As the tests showed normal results, the patient was referred to magnetic resonance imaging (MRI) of the cervical spine and X-ray of the left palm. The X-ray findings were normal, while the MRI demonstrated subtle signs of C5–C6 level compressive radiculopathy. The patient underwent an MRI of the left elbow and left wrist in September 2016. Indirect signs of ulnar nerve neuropathy in the cubital canal were registered—hyperintense nerve signal on T2 and STIR sequences. Instability of the central carpal area was also noted. The EMNG, performed in July 2017, showed no pathology progression in comparison to the earlier one, without clear-cut criteria matched for focal ulnar neuropathy. Finally, as the symptoms did not improve, X-ray images of the patient’s shoulder were obtained at the end of June 2018 (Figure 1). Within the next month, multi-slice computed tomography (MSCT) and MRI of the left shoulder were obtained. MSCT of the left shoulder confirmed the presence of multiple osteolytic lesions with lamellar periosteal reaction and sporadic cortical breaches in the left scapula. MRI of the left shoulder (Figure 2) confirmed the suspicion of a neoplastic process of the left scapula and the need for further orthopedic and diagnostic evaluation. An orthopedic examination revealed a normal range of motion of the left shoulder and positive signs of ulnar nerve compression in the cubital canal of the elbow. Scintigraphy showed an intensified technetium-99 m accumulation in the left scapula. An incisional biopsy of the suspicious lesion was performed at the end of August 2018. Sample morphology demonstrated neoplastic tissue erected from large, atypical, lymphatic cell-type centroblasts. Immunohistochemically, tumor cells were positive for markers such as the cluster of differentiation (CD) CD20, CD10, and B-cell lymphoma 6 (BCL6), C-Myc was positive in around 35% of tumor cells, and Ki-67 was 80%. Biopsy findings confirmed the clinical diagnosis of non-Hodgkin lymphoma (NHL), diffuse large B-cell lymphoma (DLBCL), germ-cell phenotype (GCB), stage IV. The patient did not have the “B” symptoms. Treatment with rituximab, cyclophosphamide, doxorubicin, vincristine, and methylprednisolone (R-CHOP) immunochemotherapy started at the end of September 2018. Bisphosphonate zoledronic acid (ZA) for the prevention of bone resorption was given monthly for the next two years. Because of the positive hepatitis B virus (HBV) serology, specifically anti-HBs (hepatitis B surface) antibody, total anti-HBc (hepatitis B core) antibody, and anti-HBe (hepatitis B e antigen) antibody, lamivudine was introduced during R-CHOP treatment to prevent HBV reactivation for one year. Interim Positron Emission Tomography-CT (PET-CT), performed in January 2019, showed areas of mixed sclerotic and osteolytic lesions with slightly higher metabolic activity in the left scapula. No lymphadenopathy was observed. After six cycles of CHOP chemotherapy (final at the end of January 2019) and eight doses of rituximab immunotherapy (final in April 2019), the end-of-treatment PET-CT was performed in May 2019. It showed no differences compared to the previous PET-CT. Radiotherapy of the left scapula with 30 Gy in 15 fractions was completed in July 2019.

The patient continued follow-up at the outpatient clinic. Light paresthesia and tingling in feet and hands subsided in the period between the two follow-ups. A control PET-CT was repeated in November 2019. There was no pathological metabolic activity of the fluorodeoxyglucose. After one and two years, the patient terminated lamivudine and ZA consecutively. The patient had a follow-up by a neurologist in October 2020 due to paresthesia in both forearms and lower legs. The physical exam presented the loss of sensation for the crude touch and temperature in the distal parts of the legs and arms. The sensation of vibration was preserved, and no accompanying motoric weaknesses were present. Clinical diagnosis of polyneuropathy was determined, presumably toxic neuropathy, mostly due to vincristine. An EMNG was ordered for the patient, which showed some possible signs of smaller, myelinated fiber neuropathy. The patient refused therapy for his mild neurologic symptoms. During the regular follow-up in May 2021, the patient reported right axillar lymphadenopathy. He underwent ultrasound-guided fine needle aspiration cytology, which showed reactive proliferation. The lymphadenopathy appeared 2 weeks after the coronavirus disease 2019 vaccine, and a few weeks later, it was completely resolved. The patient also had an exam by an orthopedic specialist because of straining and tightening in the left shoulder when he was physically active. Although significantly less pronounced than before hematologic treatment, paresthesia and tingling in the little finger of the left hand were still present. The function of the shoulder was minimally affected, as there was only terminal restriction of external rotation and slightly decreased strength of internal and external shoulder rotation. Noteworthy information from the patient’s history is Baker cyst and pigmented villonodular synovitis of the right knee. More than twenty years ago, an open synovectomy of the right knee was performed. Despite the remission, during mid-2023, the patient developed right-sided hemiparesis and sensory impairment with the diagnosis of ischemic stroke. Acetylsalicylic acid and atorvastatin were introduced in therapy. Afterward, the patient successfully passed the rehabilitation period, with significant recovery in motor and sensory function. The patient is currently in the process of evaluation for thrombophilia. Also, the patient underwent a transcranial Doppler bubble test, which came back positive, indicating the existence of a patent foramen ovale. Further cardiac assessment is being planned.

## 3. Discussion

Regardless of atypical primary non-Hodgkin lymphoma of the bone localization—scapula without lymph node involvement and unusual presentation—appropriate treatment (R-CHOP + radiotherapy) in addition to lamivudine for HBV and bisphosphonates for prevention of bone resorption resulted in complete remission of DLBCL without concomitant complications.

DLBCL is one of the most common types of NHL, with an incidence of 5.5/100,000/year [2]. A broad spectrum of risk factors for DLBCL development includes genetics, family or personal history of lymphoma, autoimmune pathology, infections, and immunosuppressive medications [3,4]. Aside from our patient, who had asymptomatic HBV infection, to our knowledge, there have been only two other reported cases of DLBCL in the shoulder area with known underlying risk factors [5,6]. The risk factors included human immunodeficiency virus (HIV) infection in the first and rheumatoid arthritis in the second case.

Most DLBCLs originate in lymph nodes, but ≤40% are of extranodal origin [1]. The most common site of extranodal origin is the gastrointestinal tract, but other organs such as testicles, the central nervous system, and bones can also be affected. The most common osseous localizations of extranodal DLBCL are femur, pelvic bones, and spine. To our knowledge, there were only five cases of primary extranodal DLBCL [7,8,9,10,11] without regional lymph node involvement located in the osseous area of the scapula (Table 1).

It was found that patients treated for DLBCL usually wait for 4 weeks before they seek medical attention, and it is 8 weeks before non-hematology physicians diagnose DLBCL and refer them to a hematologist [12]. Patients with DLBCL localized in the scapula mostly presented with shoulder pain and localized swelling that is consistent with the usual presentation of primary bone lymphoma [13]. Although it may not be a direct consequence of lymphoma, the unusual clinical presentation with paresthesia and edema of the little and ring fingers on the left hand is unique among patients with osseous DLBCL in the shoulder region. The shortest distance between the DLBCL infiltrate and the distal part of the brachial plexus in our patient was measured to be 33 mm (Figure 2C,D). Due to such a long distance, it is hard to correlate the patient’s neurologic symptoms with the DLBCL infiltration, but the fact that symptoms significantly decreased following hematologic treatment remains.

Despite the slightly prolonged time to the diagnosis, this did not affect the success of treatment in our patient. Other than the cell of origin classification, an array of prognostic factors that, if present, are associated with poor outcome include higher age (>60 years), immunoblastic subtype, presence of pathological fracture, and high-grade B-cell lymphomas [14]. Our patient has had a National Comprehensive Cancer Network International Prognostic Index score of 2, which means he belongs to a low-intermediate risk group with an estimated 5-year progression-free survival of 74% and an estimated 5-year overall survival of 82%.

Despite the patient lacking typical local symptoms such as pain and swelling, biopsy showed large, atypical cells and immunohistochemical staining was positive for markers CD10 and CD20, pointing to lymphoma. Differential diagnosis of any bone lesion includes a broad spectrum of conditions such as chronic osteomyelitis, primary bone lymphoma, either primary or metastatic osteosarcoma, Ewing sarcoma, chondrosarcoma, metastasis, etc. Clinical history and different types of diagnostic imaging can sometimes help differentiate between bone lesion etiologies, but biopsy and immunohistochemical staining have an unavoidable role in diagnosis confirmation [15]. Any bone lesion detected in patients above 40 years old must be ruled out for metastasis and myeloma [16]. The prominent periosteal reaction in our patient was in contrast with earlier findings, which associate a higher degree of bone destruction and periosteal reaction with osteosarcoma and Ewing sarcoma [17]. As a result of treatment, persisting bone changes during follow-ups caused difficulty in differentiating between active, malignant disease and bone healing. Signal abnormalities on MRI may persist for up to 2 years, while bone remodeling seen on CT has similarities with Paget’s disease [18]. In the case of interim PET-CT positive findings, repeated biopsy of residual masses should be considered [19].

Approximately 20–50% of patients with HBsAg positivity and 3–45% of patients with HBcAb positivity develop HBV reactivation following chemotherapy [20]. The risk for reactivation is twofold higher if patients are managed on rituximab compared to chemotherapy alone [21]. Although our patient received lamivudine therapy pre-emptively, it is no longer the main therapy choice due to a rise in viral resistance, and more specific anti-viral drugs, such as entecavir, are recommended [22]. Our patient continued receiving lamivudine for one year, which is in line with the current guideline recommendations [23]. Two questions concerning such lamivudine application are the risk of HBV reactivation after the lamivudine withdrawal and HBV DNA mutation induction, which can cause a hepatitis flare [24]. Neither occurred in the patient following cessation of lamivudine treatment.

In addition to the antiresorptive effect, ZA has a potential antiangiogenic effect by decreasing basal serum vascular endothelial growth factor for the next three weeks and decreasing platelet-derived growth factor for the next two days [25]. Its anti-tumor effect is not so distinctive. ZA was able to induce tumor cell apoptosis in vitro, but concentrations needed for that were 10–100× higher than those used in osteoclast apoptosis induction in vitro and in vivo. Another impediment to ZA’s anti-tumor effect in extra-skeletal sites is its pharmacokinetics—rapid elimination from plasma resulting from renal excretion and rapid uptake and accumulation within bone [26]. A relatively recent study performed on patients with DLBCL and bone metastasis exhibited no difference in OS, progression-free survival, and complete remission rate in patients treated with ZA compared to the control group [27]. On the other hand, the systematic review and meta-analysis of ZA in patients with metastatic breast cancer concluded that ZA use as adjuvant therapy has a significant impact on the patient’s overall survival and lower fracture rate. The positive outcome was not present after ZA usage for disease-free survival compared to the control group [28]. Currently, the standard interval dosage is 4 weeks. The research conducted a few years ago indicated no difference in pain occurrence or skeletal-related events when patients received bisphosphonate therapy every 12 weeks compared to the standard 4-week interval [29].

Despite earlier studies showcasing the connection between NHL and hypercoagulability [30], it is highly unlikely that lymphoma was the primary cause of ischemic stroke in our patient due to a substantial remission period between events.

## 4. Conclusions

In conclusion, primary neurological symptoms that cannot be explained by neurological and radiological examinations require further investigation of the locomotor system. In the case of underlying lymphoma, a combination of chemoimmunotherapy and radiotherapy is related to high complete remission rates.

## Figures and Tables

**Table 1:** Patients with extranodal diffuse large B-cell lymphoma (DLBCL) localized only in scapula listed by the year of publication.

1st Author and Year of Publication	Sex/Age	Presentation	Time between Symptom Onset and Diagnosis/ Time between First Hospital Visit and Diagnosis	Involvement of Other Sites	Treatment	Outcome
Chauhan [7], 2013	F/84 y	Pain	Two months/ days-weeks	Surrounding soft tissue	R-CHOP chemotherapy	Remission
Carroll [8], 2013	M/29 y	Lump	Nd/Nd	Nd	Nd	Nd
Boulytcheva [9], 2013	F/73 y	Nd	Nd	Nd	Radiotherapy	Death
Das [10], 2017	M/73 y	Nd	Nd	Nd	Nd	Nd
Ayesh Haj Yousef [11], 2022	F/46 y	Mass	Two months/Nd	Nd	R-CHOP chemotherapy + radiotherapy	Remission
Our patient	M/45 y	Paresthesia, tingling, numbness and edema of the little and ring fingers	Two years/ two years	Surrounding soft tissue	R-CHOP chemotherapy + radiotherapy	Remission

Note: F—female; M—male; Nd—not disclosed; y—years; R-CHOP—rituximab, cyclophosphamide, doxorubicin, vincristine, and methylprednisolone.

## Data Availability

The data used and analyzed during the current study are available from the corresponding author on reasonable request.
